# Nurses’ perceptions towards the delivery and feasibility of a behaviour change intervention to enhance physical activity in patients at risk for cardiovascular disease in primary care: a qualitative study

**DOI:** 10.1186/s12875-018-0888-1

**Published:** 2018-12-12

**Authors:** Heleen Westland, Yvonne Koop, Carin D. Schröder, Marieke J. Schuurmans, P. Slabbers, Jaap C. A. Trappenburg, Sigrid C. J. M. Vervoort

**Affiliations:** 1 Julius Center for Health Sciences and Primary Care, University Medical Center Utrecht, HP Str. 6.131, PO 85500, 3508 GA Utrecht, The Netherlands; 2 Department of Cardiology, Radboud University Medical Center, Nijmegen, The Netherlands; 3 Center of Excellence in Rehabilitation Medicine, Brain Center Rudolf Magnus, University Medical Center Utrecht, University Utrecht and De Hoogstraat Rehabilitation, Utrecht, the Netherlands; 4 Department of Acute Psychiatry, Psychiatric Center GGZ Central, Amersfoort, The Netherlands; 5 Cancer Center, University Medical Center Utrecht, Utrecht, The Netherlands; 6 Education Center, UMC Utrecht Academy, University Medical Center Utrecht, Utrecht University, Utrecht, The Netherlands

## Abstract

**Background:**

Self-management support is widely accepted for the management of chronic conditions. Self-management often requires behaviour change in patients, in which primary care nurses play a pivotal role. To support patients in changing their behaviour, the structured behaviour change Activate intervention was developed. This intervention aims to enhance physical activity in patients at risk for cardiovascular disease in primary care as well as to enhance nurses’ role in supporting these patients. This study aimed to evaluate nurses’ perceptions towards the delivery and feasibility of the Activate intervention.

**Methods:**

A qualitative study nested within a cluster-randomised controlled trial using semistructured interviews was conducted and thematically analysed. Fourteen nurses who delivered the Activate intervention participated.

**Results:**

Three key themes emerged concerning nurses’ perceptions of delivering the intervention: nurses’ engagement towards delivering the intervention; acquiring knowledge and skills; and dealing with adherence to the consultation structure. Three key themes were identified concerning the feasibility of the intervention: expectations towards the use of the intervention in routine practice; perceptions towards the feasibility of the training programme; and enabling personal development.

**Conclusions:**

Delivering a behaviour change intervention is challenged by the complexity of changing nurses’ consultation style, including acquiring corresponding knowledge and skills. The findings have increased the understanding of the effectiveness of the Activate trial and will guide the development and evaluation of future behaviour change interventions delivered by nurses in primary care.

**Trial registration:**

ClinicalTrials.gov NCT02725203.

**Electronic supplementary material:**

The online version of this article (10.1186/s12875-018-0888-1) contains supplementary material, which is available to authorized users.

## Background

Self-management support is widely accepted as an approach to improve health-related outcomes, enhance patients’ involvement and decrease healthcare costs [1–3]. Self-management support by health care providers, such as primary care nurses, aims to equip patients with the essential skills to manage symptoms, treatment, physical and psychosocial consequences of chronic diseases and to change patients’ health behaviour [4, 5]. Over the past decade, in most Western countries, disease management of some of the most prevalent chronic conditions, including diabetes mellitus type 2 and (risk of) cardiovascular disease (CVD), has shifted away from hospitals and towards primary care. In primary care, chronic care is increasingly reallocated from general practitioners towards primary care nurses [6]. Primary care nurses play a pivotal role in the management of chronic conditions, promoting self-management and offering follow-up consultations [6]. Therefore, they in a key position to support these patients in changing their health behaviour [6]. Like other behavioural interventions, self-management interventions are considered complex, containing multiple interacting components [7]. Self-management support requires nurses to adapt their traditional consultation style, which is focused on giving advice, informing and educating patients about their condition, towards a more coaching-oriented consultation style aimed at supporting patients in changing their behaviour [8–10]. Adapting their consultation style adequately implies that nurses need to change their behaviour, which is challenging to accomplish [8, 11–14]. Furthermore, in order to change and incorporate their adapted consultation style into their routine practice, nurses need to be facilitated and supported by their superiors, for instance through being autonomous, having enough time to integrate self-management into their consultations and having training opportunities [11, 12]. The effectiveness of self-management interventions is often evaluated in randomised controlled trials that are mainly focused on pre-specified outcomes rather than on in-depth exploration of the delivery and implementation process [15]. Insight into the perceptions of providers towards the delivery and feasibility of such interventions, as part of a process evaluation, might enhance our understanding of the effectiveness of complex interventions and shed some light on how the intervention works [16–19].

This study evaluated the perceptions of the providers towards the delivery and feasibility of a self-management intervention alongside the cluster-randomised controlled Activate trial. The Activate intervention is a nurse-led behaviour change intervention targeted at increasing physical activity in a large heterogeneous subgroup of patients, namely, those at risk for CVD. The research questions of this study were:

1. What are primary care nurses’ perceptions of delivering the Activate intervention to patients at risk for CVD?
2. What are primary care nurses’ perceptions of the feasibility of the Activate intervention for routine practice?

## Methods

### Study design

A qualitative study of nurses’ perceptions of delivering the Activate intervention, nested within a cluster-randomised trial in primary care, was conducted.

### The Activate intervention

To enhance behaviour change in both patients and nurses, the Activate intervention was developed using the Behaviour Change Wheel (BCW) [20]. A behavioural analysis was conducted for the behaviour of patients and the behaviour of nurses using the COM-B (capability, opportunity, motivation-behaviour) model [20]. Subsequently, intervention functions were selected, by which patients’ level of physical activity and nurses’ skills to provide support could be enhanced. The intervention functions were linked to a selection of behaviour change techniques (BCTs) to support behaviour change [20, 21].

Behavioural analysis of the patients resulted in a selection of 17 BCTs, which were integrated into the Activate intervention. The intervention consisted of four standardised nurse-led consultations to enhance physical activity spread over a 12-week period: one consultation in the first week with subsequent consultations after 2, 6 and 12 weeks. Consultations occurred in the patients’ own general practice, with a duration of 20–30 min.

The intervention structure was described in a handbook for nurses. Nurses were asked to individualise the content of the consultations to the patients’ unique circumstances, needs and preferences. Patients received a workbook, which included tips and tricks, useful websites, activity logs and action plans and were equipped with an accelerometer (personal activity monitor; Pam AM300) [22] in order to self-monitor their physical activity daily.

Behavioural analysis of the nurses resulted in a selection of 21 BCTs, which were integrated into a standardised comprehensive training programme to equip nurses with the skills to deliver the Activate consultations to patients. The training consisted of several components: a one-day training, two individual coaching sessions, instructional videos on how to apply the BCTs in the consultations, a handbook with example sentences and checklists (what to do when). Preparatory to the one-day training, nurses received a workbook, including study procedures and materials and were asked to view two online presentations to reinforce the procedures and the relevance of physical activity for patients at risk for CVD. The one-day training was held in a small group led by a health psychologist, and it focused on learning how to deliver the BCTs in each of the consultations. This training included theoretical background about how to promote behaviour change and included practising skills in delivering the consultation using an outlined structure, which included BCTs, by use of instructional videos and role-playing. To optimise and rehearse the gained skills, nurses received two individual coaching sessions by the health psychologist. For each coaching session, nurses recorded one of their consultations on which they received feedback on their performance during the coaching session. To strengthen their gained skills, nurses were encouraged to use the instructional videos, handbook and checklists.

Further details on the development and content of the intervention are described elsewhere [23].

The Activate intervention is currently being tested for its effectiveness in terms of number of minutes of moderate to vigorous physical activity within a 6-month follow-up period in a two-armed cluster-randomised controlled trial in primary care settings in the Netherlands comparing the Activate intervention with care as usual, according to the Dutch guideline of cardiovascular risk management. The Activate trial entails participation by 31 general practices, 36 primary care nurses and 195 patients (Activate trial, ClinicalTrials.gov NCT02725203). A total of 16 general practices (20 primary care nurses) were randomly allocated to the intervention group and a total of 15 general practices (16 primary care nurses) were randomly allocated to the control group.

### Sample and recruitment

The study sample consisted of 20 primary care nurses from 16 general practices situated throughout the Netherlands who participated in the Activate trial and were allocated to the intervention group. Nurses were eligible to participate if they had experience with delivering the intervention, which was operationalised as having completed the training and delivered the intervention to at least two patients. Therefore, two nurses were excluded from this study, as they had delivered the intervention to fewer than two patients due to difficulties recruiting patients. One nurse was excluded because she had changed jobs during the study. After completing the intervention, all eligible nurses (*n* = 17) were invited through email to participate in this qualitative study. In total, 14 nurses (82.4%) agreed to participate, and 3 nurses refused to participate due to busy clinical practice.

To increase the likelihood of reflecting different nurse perspectives and to increase the representativeness of the data, maximum variation sampling was used in the recruitment phase of the Activate trial to obtain diversity with regard to nurses’ age and years of working experience with patients at risk for CVD in primary care. Furthermore, we strived for maximum variation in the sample with regard to nurses’ educational background, as some nurses -other than working as a registered nurse- had formerly worked predominantly as receptionists and practitioner assistants in general practices prior to their specialisation in primary care nursing.

### Data collection

Face-to-face individual interviews were conducted using a semi-structured interview guide. This consisted of open questions asking about perceptions towards the training, intervention delivery, effect on patients’ behaviour, changes in consultation style and feasibility of the intervention in practice (Additional file 1). Based on nurses’ narratives, topics that were mentioned were explored in depth. The interview guide was developed by four researchers and peer reviewed by the research team to ensure feasibility and completeness of the topics. All interviews started with the same opening question: “What was the reason you agreed to participate in the Activate study?”

The interviews were conducted by three researchers. An expert on qualitative research was involved in the process to ascertain the methodological quality of the study.

The interviewers were unknown to the nurses, enabling them to express their experiences and opinions without inhibitions. Nurses were interviewed once at the general practice or at the nurses’ homes based on nurses’ preferences. Interviews ranged in duration from 35 to 62 min (mean: 48 min). All interviews were audio-recorded.

During and after the interviews, memos were made to describe observations, reflect on methodological issues, capture initial ideas about emerging themes and inform refinements of the interview guide. Furthermore, the interview techniques of the interviewers were discussed and they were trained by the research team to ameliorate the equivocality of the interviews. Nurses’ baseline characteristics were collected in the Activate trial.

Ethical approval to conduct the interviews was awarded within the overall approval for the Activate trial, which was approved by the Medical Ethics Research Committee of the University Medical Center Utrecht (NL54286.041.15).

### Data analysis

All interviews were transcribed verbatim. Data were thematically analysed [24]. Data analysis started after the first three interviews. The transcripts were read and re-read, initial ideas for coding and refinements of the interview guide were discussed. After every three interviews, the transcripts were double-coded and the codes were assessed for similarities and differences by the research team. Subsequently the initial codes were collated into potential themes, and all relevant data were structured to each potential theme. Potential themes and subthemes were reviewed on consistency with the codes and entire data to ensure they reflect the entire data. Inconsistencies were discussed during joint meetings with the research team and themes were further developed and depicted in a thematic map of the data. Furthermore, the essence of each theme was further considered by the research team, themes were defined and illustrative quotes were selected.

Data saturation was reached after the twelfth interview; however, the data were complemented with two interviews to affirm the potential themes and ensure a maximum variation in the sample.

Data analysis was supported by NVivo 11.0 software (QSR International Pty Ltd., Version 11.0, 2011).

### Trustworthiness

Credibility of data collection and analysis was enhanced by researcher triangulation and peer review in all phases of the study [25]. An expert on qualitative research was involved in the process to ensure accuracy and enhance data dependability [26]. Biweekly meetings with four team members to discuss data collection and analysis decisions enhanced methodological quality. In addition, an audit trail ensured the study’s confirmability [25]. Memo writing and expert opinion supported the analysis and enhanced study reliability [26]. The use of a 15-point checklist by Braun and Clarke [24] ensured correct application of the phases of thematic analysis; see Additional file 2. The consolidated criteria for reporting qualitative studies (COREQ) were used to facilitate reporting of the results [27]; see Additional file 3.

## Results

Between October 2016 and March 2017, 14 nurses were interviewed. All nurses were female. Maximum variation was achieved for age (range 24–63 years; mean 48.9), years of experience working with patients at risk for CVD in primary care (range 2–14 years; mean 7.2) and educational background (*n* = 11; 73.3% registered nurses). Nurses’ characteristics are presented in Table 1.

**Table 1:** Characteristics of participating primary care nurses

ID	Age range	Working experience (years)^a^	Educational background	Additional training	Included patients in the study (n)
R1	51–55	12	Former practice assistant	None	2
R2	41–45	14	Former practice assistant	MI, SQ	10
R3	61–65	2	Former practice assistant	None	3
R4	51–55	5	Registered nurse	MI	3
R5	51–55	5	Registered nurse	MI	11
R6	46–50	9	Registered nurse	None	5
R7	36–40	9	Registered nurse	None	10
R8	36–40	2	Registered nurse	MI	2
R9	56–60	2	Former practice assistant	MI	5
R10	51–55	11	Registered nurse	MI	7
R11	56–60	6	Former practice assistant	MI	5
R12	46–50	13	Registered nurse	MI, SQ	2
R13	21–25	3	Registered nurse	MI	3
R14	51–55	8	Registered nurse	MI, SM	5

*Abbreviations: CVD* cardiovascular diseases, *MI* motivational interviewing, *SM* self-management, *SQ* Socratic Questioning

^a^Working experience as a nurse in primary care with patients at risk for CVD

**Fig. 1:**
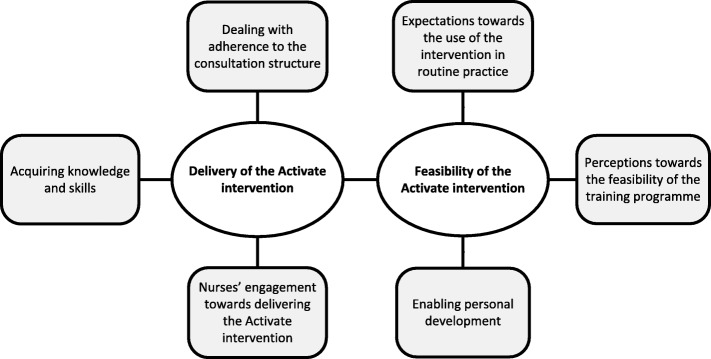
Thematic map of nurses’ perceptions of delivering and the feasibility of the Activate intervention

A thematic map was created to depict the emerged themes; see Fig. 1. Three themes emerged in answer to research question 1: nurses’ perceptions towards delivering the Activate intervention:

- Nurses’ engagement towards delivering the Activate intervention
- Acquiring knowledge and skills
- Dealing with adherence to the consultation structure

Research question 2: nurses’ perceptions towards the feasibility of the Activate intervention for routine practice, was captured in three themes:

- Expectations towards the use of the intervention in routine practice
- Perceptions towards the feasibility of the training programme
- Enabling personal development

### Nurses’ engagement towards delivering the activate intervention

All nurses indicated that contributing to the improvement of patients’ health outcomes was the core of their nursing role, which aligned with delivering an intervention to enhance patients’ behaviour change. Reasons for participating in the Activate trial corresponded with their beliefs about the advantage of increased physical activity for lowering the risk of CVD and thus improving health outcomes. Based on their experience in supporting patients to change their health behaviour, all nurses expressed a need to increase their skills to enhance their support to patients in order to increase physical activity.

*“The main reason was that it's difficult to motivate people to increase their physical activity. I could use some tools for how I could handle this the best way. Very often, questions about patients’ motivation remain superficial, and I wanted to know how I am going to ask in-depth questions about their motivation?” (R10)*

Directly after the training, all nurses felt engaged to deliver the intervention in their practice. Their engagement was supported by having been convinced that the intervention could be beneficial to patients.

*“I felt that I could perform better in my job, that I could make a difference to people and that I have more to offer them.” (R6)*

Nurses expressed that, during the study period, their engagement towards delivering the intervention strongly depended on their experiences with delivering the intervention.

All nurses valued and felt rewarded by patients’ success at increasing their level of physical activity and perceived this as an effect of their intervening activities. Patients’ success had a positive impact on the nurses’ job satisfaction and their engagement towards delivering the intervention.

*“It is very nice to see that it just has an effect on people and that people feel fitter. That makes you excited and willing to continue. Ultimately, it is what you want to do: to help people further.” (R13)*

However, differences were seen in how nurses dealt with patients’ lack of motivation to participate in the intervention or their lack of success at increasing their physical activity, and this negatively affected the engagement of some nurses. Patients’ lack of motivation to participate often led to a postponed start for delivering the intervention or fewer practising opportunities. Some nurses felt rewarded by enhancement of their knowledge and skills to support patients, in particular with patients who they perceived as challenging to motivate. They perceived that actual delivery of the intervention increased their confidence and job satisfaction and helped them to positively continue with the study despite perceived difficulties with patient inclusion.

*“Otherwise, you have nothing to discuss, right? If someone has 100% perseverance, then you are soon done. You have a lot more to discuss, that’s nice. If someone says, ‘I have already tried it ten times, but I can’t keep it up’, well then you have something to look for.”(R12)*

Despite their high initial engagement, some nurses felt that their engagement towards delivering the intervention strongly depended on patients’ motivation. Patients’ unwillingness to participate as well as patients’ lack of commitment to goal attainment resulted in some nurses questioning their efforts to support them, which affected their engagement negatively.

*“For me, it's more fun to support a motivated patient who does his homework perfectly compared to a patient who brings a completely empty diary and says, ‘Yes, I did not really keep up’. Then, you think this costs me forty-five minutes, and that patient actually does not do anything. It's a lot more fun when they say, ‘I deliberately went cycling to reach my goal.’ Yes, then you really feel like that’s what I am doing it for.” (R2)*

While continuing their participation in the study, nurses had to deal with circumstances such as a high work load and absence due to sick leave or holiday, which often negatively influenced their engagement towards delivering the intervention.

### Acquiring knowledge and skills

All nurses reported that the training, handbook, checklists, instructional videos and coaching sessions were essential to equip them with the necessary knowledge and skills to establish and deliver the intervention as intended, which strengthened their confidence and engagement in their support.

*“After the training, I felt I had a lot of tools I could apply to patients. I was equipped with a lot of techniques for gaining effects in patients, and that feels good. Normally, I asked: ‘are you physically active?’ Now, I make it more specific and explore with the patient how to continue.” (R6)*

To strengthen their confidence, nurses reinforced their gained skills and knowledge by rehearsing the consultation structure using the instructional videos and the handbook.

Once their initial feelings of uncertainty towards their skills were overcome and their confidence improved, brief repetition of the handbook prior to a consultation was sufficient.

*“I really regretted there was a long delay between the training and the first consultation. Then, things dwindled pretty fast since you are not practising it. Only prior to my first and second patient I watched the instructional videos again. Then, I had the idea that I had a better grip on it.” (R6)*

Most nurses felt regular practice was the most beneficial factor for developing their skills; however, some nurses felt a need for additional training with the health psychologist to refine their skills.

Furthermore, focusing solely on physical activity without getting in conflict with other clinical demands, enabled them to develop their skills.

*“This sure is special, whereas you normally don’t do this, since there is a lot more in a consultation. But, yes, you notice that once you have more time, you can practise a lot.” (R10)*

Nurses’ participation in the intervention, in particular the role-playing and coaching, exposed their habits with regard to their own consultation style and skills, such as solving patients’ problems by giving advice and filling in for the patient. Once nurses became aware of their habits, they identified that changing their routines by applying the acquired knowledge and skills was challenging, as they easily fell back into their traditional style.

*“Sometimes, I noticed I was the one searching for solutions. Of course, that was not how it was meant. That's very typical for nurses’ way of doing things. Then I thought, well, I’m sitting here working, while the one in front of me should work.”(R14)*

*“The feedback from the coach was a kind of eye-opener; I do things, but I did not ask in-depth questions…that made me think and I took that with me to the next consultation. I found that to be particularly useful.” (R12)*

Nurses valued that they could transfer their developed skills to other patients and to other behaviours, such as smoking cessation and dietary intake. This indirect benefit enhanced nurses’ engagement to deliver the intervention.

*“Now, it is very much focused on physical activity, but I think, in any case, helping people with behavioural change is something that you can see broader applications, like for other lifestyle topics.” (R12)*

### Dealing with adherence to the consultation structure

To ensure fidelity of the intervention, nurses were aware that they had to adhere to the structure of the consultations as described in the handbook, even if they had personal doubts about specific elements. However, some nurses deliberately deviated from the consultation structure when they were not convinced of the effectiveness of a specific element or when they did not feel comfortable with an element. Furthermore, most nurses valued the use of the handbook in their consultations, as this allowed them to follow the structure and use example sentences more easily. Nurses often changed the wording of the sentences to something which they felt more comfortable with.

*“There was a question about patients’ confidence, which didn’t make me very happy. But it is part of the intervention, I know. I have tried to ask it.”(R12)*

*“But you don’t talk like these sentences. I make my own sentences. But, yes, of course it helps. You don’t literally say it like that though. Because then…the conversation is less fluent.” (R1)*

After the training, most nurses reported that adhering to the consultation structure was more difficult than expected, which reduced their confidence in their capabilities. Patients easily initiated other topics, as they were used to doing so in the routine consultations. That distracted the nurses from following the prescribed structure.

*“I sometimes found it difficult to follow the script, prompting me to think, well, this is yet more difficult than I thought. So, then, my confidence decreased. I can certainly understand how it works on paper and that it works, but in practice it’s different.” (R8)*

To enhance fidelity of the intervention, nurses were aware that they had to fill in the charts at the end of each consultation to check if they discussed all of the elements described in the handbook.

### Expectations towards the use of the intervention in routine practice

Nurses’ beliefs about the use of the intervention in their routine practice strongly depended on their beliefs about the effectiveness of the intervention to increase patients’ level of physical activity and health outcomes. Nurses were convinced that the effectiveness of the intervention relied on patients’ engagement to set goals and having a reasonable level of health literacy to understand the intervention materials.

*“It works in patients who just need a helping hand to perform it. But the truly unmotivated patients who don’t want to be active, those patients are not going to be active using this method, no. They still have to do it themselves.” (R2)*

The nurses were convinced that the combination of the accelerometer, activity log and their subsequent and structural support incentivised patients’ goal attainment in changing their physical activity, which strengthened their positive beliefs about the feasibility of the intervention in their routine practice.

*“If you would send them home with an activity log but without consultations, then no one would fill it in. But now they have to come back. Then they must do it anyway, because of course they know it will be discussed then…I found the activity log was very good. Patients confirmed that. However, so were the consultations. So, basically, just the combination really made it work.” (R2)*

Nurses valued the consultation structure, including techniques such as goal setting, action planning, reviewing behavioural goals, feedback on behaviour, self-monitoring and problem solving, as being feasible to use in their routine practice. Most nurses found that goal setting and action planning enabled them to stimulate patients in formulating their goals and actions, which in turn facilitated patients’ goal attainment. The use of the activity log to review patients’ level of goal attainment facilitated them in giving feedback on their behaviour.

*“You have to make it specific; otherwise, it won’t work. If you make it very specific, patients also know: all right, that's my goal and here I go. And then you can say, ‘I've done it or not’ ... I was always aware of the fact that patients specified their planned actions. If patients said, ‘I'm going to be active in five days’, that’s, of course, not very specific, so I tried to make it even more specific.” (R13)*

The use of self-monitoring tools such as the accelerometer and activity log were seen as additional motivators and incentives for patients, as they provided insight into patients’ level of physical activity and challenged patients to goal attainment. The nurses believed that the use of such tools would help them to deliver the intervention in their routine practice.

*“The accelerometer just provides insight, which makes your activity very specific. Actually, you normally don’t really think about it that much.”(R13)*

Despite the log and accelerometer being highly valued by nurses, a few nurses questioned the usability of such tools in their routine practice as some patients did not completely understand the user instructions and faced practical and technical problems, such as losing the accelerometer or losing their activity data after the accelerometer automatically reset at midnight.

*“I noticed that it was quite complicated for patients…the accelerometer was difficult to operate…And the fact that the accelerometer erased itself after midnight, then they couldn’t read it out anymore.” (R9)*

Although all nurses believed that the intervention was feasible for routine practice, they thought that using the intervention in routine practice might conflict with other clinical demands during routine consultations. Initially, nurses needed more time to deliver the intervention, which may adversely influence the feasibility due to time constraints in their routine practice. However, nurses believed that mastering the necessary skills would enable them to gain more in-depth support, which eventually would save them time. To enhance the fit of the intervention in routine practice, nurses suggested shortening the number of in-depth questions.

*“I think it takes too much time to do it in such an extensive way. You also need to check patients and discuss their medications, insulin and whatever.” (R14)*

*“It may seem like it's very time consuming, but once you ask the right questions then I think you can get a lot of information in a short period of time, and it's a bit of an art to let the patients talk themselves.” (R10)*

### Perceptions towards the feasibility of the training programme

All nurses felt appropriately trained and supported by the one-day training in combination with the instructional videos, handbook and checklists to deliver the Activate intervention. The nurses particularly valued the safe learning environment of the small-scale role playing, in which they directly received feedback.

*“…first of all, the small-scale, practising with two… At least for me, it’s an obstacle to practice a role-play in front of a group… and having someone to observe… who provided feedback. So, it was a very safe setting in which, without being judged or anything, you received objective feedback.” (R11)*

Although all nurses valued the coaching, some initially felt uncomfortable submitting a recorded consultation, and delayed doing so. However, afterwards, the nurses regretted postponing their submissions, because they felt that the feedback would have helped them in delivering other consultations. Some nurses could not overcome their uncomfortable feelings surrounding recording their consultations and did not submit any consultation.

*“I just found it difficult to record it, and then it's indelible, and then you will send it, and people will listen to it. That's just a bit of an uncomfortable idea...Therefore, I was a little late with recording a consultation, which was a bit of a pity. So, I could not apply the feedback so much afterwards.” (R7)*

### Enabling personal development

All nurses expressed that participating in the Activate trial enabled their personal development and enhanced their knowledge and skills to support patients in their behaviour change. The nurses tended to incorporate specific skills and elements of the intervention into their routine practice that they were convinced were effective for patients, such as setting specific and attainable goals and planning actions for goal attainment. Nurses became more critical towards patients’ answers and used a more positive approach, focusing on solutions instead of traditionally addressing barriers for patients.

*“I became more aware of the fact that it’s important for someone to come up with their own solution, even though I am staggering with enthusiasm…if I take a step back, more can arise from oneself and that is very powerful in this work.” (R8)*

*“Specifying patients’ goal, that’s really something I've learned. And giving feedback on that goal once they come again next time. Yes, I have learned that very well.” (R6)*

## Discussion

This qualitative study explored the perceptions of primary care nurses towards delivering the Activate intervention and its feasibility in routine practice. Nurses were dedicated to deliver the intervention in order to improve health outcomes. Nurses felt engaged and rewarded by patients’ success in increasing their physical activity. Patients’ lack of motivation to participate in the intervention and lack of success negatively affected nurses’ engagement. The training, training tools and delivery of the intervention facilitated nurses in acquiring the required knowledge and skills. Acquiring skills was challenging, as the nurses tended to relapse into their traditional habits. The nurses valued and tried to adhere to the intervention structure despite perceived difficulties, such as distraction by patients who initiated discussion of topics other than physical activity.

Nurses were positive towards the feasibility of the intervention in routine practice. Nurses thought that the consultations combined with the self-monitoring tools were effective to increase patients’ physical activity and feasible to use in routine practice. However, the use of the intervention in routine practice might be hindered by complying with other clinical demands. Nurses felt appropriately trained and supported to deliver the intervention. Participation in the trial enabled their personal development and changed their routine practice, as they incorporated newly acquired skills, particularly those that they believed were efficacious, in their routine practice with other patients.

The challenges of changing nurses’ behaviour in order to enhance the implementation of behaviour change interventions are widely reported [8, 11–14, 28]. Therefore, a training programme was developed using the BCW, targeting the COM-B components using BCTs [20]. Despite the provided comprehensive training to support their patients in their own context and facilitating them with extra consultation time, changing nurses’ behaviour was complex. Delivering the intervention required nurses to shift from their traditional consultation style of being an expert, who gives advice and informs patients to a coaching consultation style that entails being supportive and facilitative to patients’ needs and preferences [8, 29]. Nurses felt comfortable in their expert role and most nurses had previously received additional training in the motivational interviewing approach; however, they unanimously expressed their need to deepen their support and increase the effectiveness of their support. This suggests that the nurses were willing to acquire the necessary knowledge and skills and to participate in the Activate trial. Participation in the trial raised awareness of their traditional consultation style and facilitated a shift to a more patient-centred approach, allowing patients to take more responsibility rather than advising and telling patients what to do, which is in line with other studies [11, 13, 29]. Despite increased awareness, it appeared difficult to perpetuate these changes in consultation style, as all nurses thought they easily relapsed into their traditional consultation style and skills, as also seen in other studies [11].

The handbook with example sentences guided nurses in structuring their consultations and facilitated their adherence towards the intervention delivery, as also seen in other studies [13, 30]. Overall, nurses tended to adjust the content of the intervention if they had personal doubts about specific elements, and this finding aligned with other studies [12, 13]. This suggests that nurses’ beliefs are pivotal with regard to the extent to which they adopt the intervention into their practice. Nurses’ tendency to tailor the intervention to their beliefs should be addressed during the training of nurses to maintain sufficient uniform delivery and underlines the need to assess nurses’ fidelity of the delivery of the intervention [13, 31]. Nurses’ engagement, confidence and job satisfaction were enhanced by patients’ success at increasing their physical activity, nurses’ personal development and transferability of knowledge and skills to other patients, as has previously been shown [11, 13, 32]. Nurses‘ job satisfaction is potentially linked to their intrinsic drive to help and assist patients. Nurses often thought that patients expect and prefer their traditional nursing role in behaviour change support. However, the patient-centred approach of the intervention requires reflection on their traditional role and adaption of their role towards facilitating and supporting patients in changing their behaviour. Changing a nurses’ role is challenging as nurses are often wedded to what they do [11, 14]. This might complicate nurses’ adoption of their gained knowledge and skills in routine practice [11].

The intervention structure and BCTs were relatively new to the nurses, as they were not specifically trained in applying and tailoring the BCTs prior to their participation in the Activate trial. Another study examining self-management support by primary care nurses in routine care found that nurses seldom focus on behaviour change and infrequently use effective techniques to support this change [33]. This strengthens the need for such training and support, because nurses are in a key position to deliver behaviour change interventions in primary care [11]. Previous studies have also found that appropriate training and support for nurses before and during delivery of the intervention is essential for the implementation of behaviour change interventions [11, 13, 32, 34]. This study showed that nurses particularly valued the small-scale role-playing in the training led by the health psychologist. The role-plays, including the feedback, allowed them to practise and directly reshape their consultation and BCT skills, and different scenarios that they perceived as difficult. This suggests that the training of nurses to deliver a behaviour change intervention should be comprehensive, interactive and delivered by a credible source, such as an expert trainer.

Despite that the nurses became more confident with their skills as they practised more often and that they were motivated to deliver the intervention as intended, they reported that recording the consultation felt uncomfortable, as they felt judged, which aligns with another study [35].

The Activate intervention included both self-monitoring tools and nurses’ support, similar to other studies [36–39]. The nurses were convinced that combining the self-monitoring tools with offering subsequent consultations was effective in changing patients’ physical activity and that the consultations were essential for enhancing patients’ engagement to continue and adjust their goals. This is in line with a study by van der Weegen et al. [36], which found that a combination of a self-monitoring tool and nurse-led consultations was effective to increase physical activity in patients with diabetes mellitus type 2 and chronic obstructive pulmonary disease. That study also found that counselling by the nurses without use of the self-monitoring tool was not effective compared to routine care.

### Strengths

This study was nested within a cluster-randomised controlled trial. Comprehensive process evaluations of complex interventions from the perspective of the providers of such interventions have been largely missing from the literature [7, 17, 18], but are increasingly being undertaken [12, 13, 34]. To prevent interpretation bias, such an evaluation should be conducted before the trial results are known. Furthermore, exploration of the perspectives of nurses may enhance implementation once the effectiveness has been established. To strengthen the trustworthiness of the study, the data were independently analysed by three researchers and supported by a qualitative research expert during the entire process. Furthermore, an audit trail, memo writing, expert opinion and the use of Braun & Clarke’ checklist [24] and the COREQ [27] enhanced trustworthiness.

The interviewers were unknown to the nurses prior to the interviews, which might have positively affected data dependability, as it allowed the nurses to express their experiences and opinions without inhibitions.

Although the results of this study were based on fourteen nurses, maximum variation sampling of nurses’ age, years of working experience with primary care patients at risk for CVD and nurses’ educational background was used to increase the likelihood of diversity with regard to nurses’ perspectives and contribute to the transferability of the results. Data saturation on all themes was achieved within these fourteen interviews, which also strengthened the transferability of the results.

### Limitations

A few limitations need to be considered. Despite all efforts to include all seventeen eligible nurses, three nurses refused to participate. Furthermore, three nurses were not eligible, as they had either used the intervention on fewer than two patients or had changed jobs during the trial. These nurses might have expressed different perspectives, which could have affected the results. The interviews were conducted at a single point in time, namely, after the nurses completed the intervention. The retrospective reflection of the nurses might not have revealed all of the individual processes with regard to delivery of the intervention and behaviour change. Furthermore, despite all efforts, for some interviews, there was a delay between the last trial consultation and the interview, potentially affecting nurses’ memory to recall. However, the researchers provided the training tools and study materials and asked further questions during the interviews to help stimulate the nurses’ memory.

### Implications

This study identified areas of concern regarding the intervention delivery and feasibility of behaviour change interventions in routine practice. First, to improve implementation, nurses need to be convinced that the intervention will be effective and is aligned with their beliefs surrounding good patient care [12]. Second, nurses must be appropriately trained according to a comprehensive training programme. Training should preferably be spread out over time, allowing and facilitating nurses to practice to refine their skills and to discuss how to address perceived difficulties, such as patient engagement and motivation to participate in the intervention. Third, to engage nurses, the developed skills should be transferable for use with other patients and behaviours. Fourth, to enhance success of the intervention, behaviour change interventions should be structured around BCTs that were highly valued by nurses, such as goal setting, action planning, reviewing behavioural goal(s), feedback on behaviour, self-monitoring, and problem solving. In addition, these BCTs are likely to be successful in changing behaviour [40–46]. Fifth, it is important for researchers and policymakers to acknowledge that adapting complex interventions on the part of providers takes time, as provider and patient behaviour change is a lengthy process [47].

## Conclusion

Delivering a behaviour change intervention is challenging as nurses have to change their traditional consultation style towards a more patient-centred consultation style. A process of acquiring and refining knowledge and skills is needed to deliver such interventions without jeopardizing treatment fidelity. Nurses were positive about delivering the intervention using a structured approach with facilitating tools and support. Comprehensive training and practising of their skills requires ongoing support to refrain from traditional habits and optimise their delivery of interventions. The nurses perceived the Activate intervention feasible in routine practice; however, incorporating the intervention into routine consultations is challenged by competing other clinical demands. This qualitative study contributes to our understanding of the complexity of changing nurses’ behaviour towards a more patient-centred consultation style. The findings can be used to enhance our understanding of the effectiveness of the Activate trial and may provide guidance for the development and evaluation of future behaviour change interventions delivered by nurses in primary care.

## Additional files

Additional file 1: Interview guide of the semi-structured interviews with nurses (PDF 184 kb)

Additional file 2: Checklist of criteria for good thematic analysis. 15-point checklist of criteria for good thematic analysis according to Braun and Clarke (PDF 303 kb)

Additional file 3: Consolidated criteria for reporting qualitative studies (COREQ). 32-item consolidated criteria for reporting qualitative studies (COREQ) (PDF 289 kb)

## Abbreviations

BCT: Behaviour change technique
BCW: Behaviour Change Wheel
COM-B: Capability/opportunity/motivation – behaviour
COREQ: Consolidated criteria for reporting qualitative studies
CVD: Cardiovascular disease
MI: Motivational Interviewing
