# Supplementary material for: Nurses’ perceptions towards the delivery and feasibility of a behaviour change intervention to enhance physical activity in patients at risk for cardiovascular disease in primary care: a qualitative study
Source: BMC Fam Pract. 2018 Dec 12;19:194. doi: 10.1186/s12875-018-0888-1 (PMC6292042; doi:10.1186/s12875-018-0888-1)
Supplement: Supplementary file 1 — Interview guide of the semi-structured interviews with nurses (PDF 184 kb) [file 12875_2018_888_MOESM1_ESM.pdf]

Additional file 1. Interview guide

Topics	Questions (examples)
General	Standard opening question: What was the reason you agreed to participate in the Activate study?
Perceptions towards the training	How did you perceive the one-day training?<br>Which component of the training helped you most/least in delivering the intervention to patients?<br>How did you perceive the instruction videos?<br>How did you perceive the role-plays?
Perceived self-confidence towards intervention delivery	After the training, how self-confident were you to deliver the intervention according to the study protocol (grade 1-10)? Did your self-confidence change during the intervention period?
Perceived motivation towards intervention delivery	After the training, how motivated were you to deliver the intervention according to the study protocol (grade 1-10)? Did your motivation change during the intervention period?
Preparing for delivering the intervention	How did you prepare yourself for delivering the study consultations?<br>Can you tell me your experiences with the handbook? To what extent did you use it?<br>Can you tell me your experiences with the charts? To what extent did you use them?<br>Can you tell me your experiences with watching the instruction videos? To what extent did you watch them?
Perceived performance of delivery the intervention	How did you perceive the individual coaching sessions by the health psychologist? To what extent did the feedback affect your performance?<br>What do you think of how you performed the intervention?<br>Which of the intervention components suited you well and which components were more difficult to deliver?
Perceived effect of the intervention on patients' behaviour	Do you think that patients benefit from the intervention? What were characteristics of patients who (not) succeeded? Which components of the study helped the patients most/least in increasing their level of physical activity?<br>Generally, how did you perceive the motivation of participating patients?
Perceived changes of consultation style	To what extent has participation in the Activate study affected your consultation style?
Focus on physical activity during consultations	How did you experience to focus solely on physical activity during the consultations?

Contact with the research team	How did you perceive the contact with the research team (accessibility, response, etc.)?
Perceived feasibility of the intervention for implementation in routine practice	Do you think the intervention could be implemented in your routine practice? Do you have any suggestions to improve the intervention for implementation in routine practice?
Additional questions regarding previous or not discussed topics	Do you have anything to add to the questions I have asked?
