# Supplementary material for: Nurses’ perceptions towards the delivery and feasibility of a behaviour change intervention to enhance physical activity in patients at risk for cardiovascular disease in primary care: a qualitative study
Source: BMC Fam Pract. 2018 Dec 12;19:194. doi: 10.1186/s12875-018-0888-1 (PMC6292042; doi:10.1186/s12875-018-0888-1)
Supplement: Supplementary file 2 — Checklist of criteria for good thematic analysis. 15-point checklist of criteria for good thematic analysis according to Braun and Clarke (PDF 303 kb) [file 12875_2018_888_MOESM2_ESM.pdf]

Additional file 2. Checklist of Criteria for Good Thematic Analysis: 15-point checklist

Adapted from: Braun, V., & Clarke, V. Using thematic analysis in psychology. *Qualitative research in psychology.2006; 3*(2), 77-101.

**Process**	**Criteria**	**Comment**
Transcription	1. The data have been transcribed to an appropriate level of detail, and the transcripts have been checked against the tapes for 'accuracy'.	Reported in manuscript
Coding	2. Each data item has been given equal attention in the coding process.	Yes
	3. Themes have not been generated from a few vivid examples (an anecdotal approach), but instead the coding process has been thorough, inclusive and comprehensive.	Reported in manuscript
	4. All relevant extracts for all each theme have been collated.	Reported in manuscript
	5. Themes have been checked against each other and back to the original data set.	Reported in manuscript
	6. Themes are internally coherent, consistent, and distinctive.	Reported in manuscript
Analysis	7. Data have been analysed – interpreted, made sense of - rather than just paraphrased or described.	Reported in manuscript
	8. Analysis and data match each other – the extracts illustrate the analytic claims.	Reported in manuscript
	9. Analysis tells a convincing and well-organised story about the data and topic.	Reported in manuscript
	10. A good balance between analytic narrative and illustrative extracts is provided.	Reported in manuscript
Overall	11. Enough time has been allocated to complete all phases of the analysis adequately, without rushing a phase or giving it a once-over-lightly.	Yes
Written report	12. The assumptions about, and specific approach to, thematic analysis are clearly explicated.	Reported in manuscript
	13. There is a good fit between what you claim you do, and what you show you have done – i.e., described method and reported analysis are consistent.	Reported in manuscript
	14. The language and concepts used in the report are consistent with the epistemological position of the analysis.	Reported in manuscript
	15. The researcher is positioned as *active* in the research process; themes do not just 'emerge'.	Reported in manuscript
