# Supplementary material for: Nurses’ perceptions towards the delivery and feasibility of a behaviour change intervention to enhance physical activity in patients at risk for cardiovascular disease in primary care: a qualitative study
Source: BMC Fam Pract. 2018 Dec 12;19:194. doi: 10.1186/s12875-018-0888-1 (PMC6292042; doi:10.1186/s12875-018-0888-1)
Supplement: Supplementary file 3 — Consolidated criteria for reporting qualitative studies (COREQ). 32-item consolidated criteria for reporting qualitative studies (COREQ) (PDF 289 kb) [file 12875_2018_888_MOESM3_ESM.pdf]

Additional file 3. Consolidated criteria for reporting qualitative studies (COREQ): 32-item checklist

Adapted from: Tong A, Sainsbury P, Craig J. Consolidated criteria for reporting qualitative research (COREQ): a 32-item checklist for interviews and focus groups. International Journal for Quality in Health Care. 2007; 19 (6): 349-57.

**No. Item**	**Guide questions/description**	**Comment**
**Domain 1: Research team and reflexivity**		
Personal Characteristics		
1. Interviewer/facilitator	Which author/s conducted the interview or focus group?	Reported in manuscript
2. Credentials	What were the researcher's credentials?	Reported in manuscript (author credentials)
3. Occupation	What was their occupation at the time of the study?	Reported in manuscript (author credentials)
4. Gender	Was the researcher male or female?	Reported in manuscript (author credentials)
5. Experience and training	What experience or training did the researcher have?	Completed a course on qualitative research
Relationship with participants		
6. Relationship established	Was a relationship established prior to study commencement?	Reported in manuscript
7. Participant knowledge of the interviewer	What did the participants know about the researcher?	Reported in manuscript
8. Interviewer characteristics	What characteristics were reported about the interviewer/facilitator?	Reported in manuscript
**Domain 2: study design**		
Theoretical framework		
9. Methodological orientation and Theory	What methodological orientation was stated to underpin the study?	Reported in manuscript
Participant selection		
10. Sampling	How were participants selected?	Reported in manuscript
11. Method of approach	How were participants approached?	Reported in manuscript
12. Sample size	How many participants were in the study?	Reported in manuscript
13. Non-participation No none participants	How many people refused to participate or dropped out? Reasons?	Reported in manuscript
Setting		
14. Setting of data collection	Where was the data collected?	Reported in manuscript

15. Presence of non-participants	Was anyone else present besides the participants and researchers?	No
16. Description of sample	What are the important characteristics of the sample?	Reported in manuscript
Data collection		
17. Interview guide	Were questions, prompts, guides provided by the authors? Was it pilot tested?	Interview guide reported in manuscript. Not pilot tested
18. Repeat interviews	Were repeated interviews carried out?	Reported in manuscript
19. Audio/visual recording	Did the research use audio or visual recording to collect the data?	Reported in manuscript
20. Field notes	Were field notes made during and/or after the interview or focus group?	Reported in manuscript
21. Duration	What was the duration of the inter views or focus group?	Reported in manuscript
22. Data saturation	Was data saturation discussed?	Reported in manuscript
23. Transcripts returned	Were transcripts returned to participants for comment and/or correction?	No because of burden to participants
**Domain 3: analysis and findings**		
Data analysis		
24. Number of data coders	How many data coders coded the data?	Reported in manuscript
25. Description of the coding tree	Did authors provide a description of the coding tree?	Reported in manuscript
26. Derivation of themes	Were themes identified in advance or derived from the data?	Reported in manuscript
27. Software What software	What software, if applicable, was used to manage the data?	Reported in manuscript
28. Participant checking	Did participants provide feedback on the findings?	No because of burden to participants
Reporting		
29. Quotations presented	Were participant quotations presented to illustrate the themes/findings? Was each quotation identified?	Reported in manuscript
30. Data and findings consistent	Was there consistency between the data presented and the findings?	Reported in manuscript
31. Clarity of major themes	Were major themes clearly presented in the findings?	Reported in manuscript
32. Clarity of minor themes	Is there a description of diverse	Reported in

	cases or discussion of minor themes?	manuscript
